# Spatially differentiated trends in urbanization, agricultural land abandonment and reclamation, and woodland recovery in Northern China

**DOI:** 10.1038/srep37658

**Published:** 2016-11-22

**Authors:** Chao Wang, Qiong Gao, Xian Wang, Mei Yu

**Affiliations:** 1 Department of Environmental Sciences, University of Puerto Rico, Rio Piedras, San Juan, PR 00936, United States of America

## Abstract

Uncovering magnitude, trend, and spatial pattern of land cover/land use changes (LCLUC) is crucial for understanding mechanisms of LCLUC and assisting land use planning and conservation. China has been undergoing unprecedented economic growth, massive rural-to-urban migration, and large-scale policy-driven ecological restoration, and therefore encountering enormous LCLUC in recent decades. However, comprehensive understandings of spatiotemporal LCLUC dynamics and underlying mechanisms are still lacking. Based on classification of annual LCLU maps from MODIS satellite imagery, we proposed a land change detection method to capture significant land change hotspots over Northern China during 2001–2013, and further analyzed temporal trends and spatial patterns of LCLUC. We found rapid decline of agricultural land near urban was predominantly caused by urban expansion. The process was especially strong in North China Plain with 14,057 km^2^ of urban gain and −21,017 km^2^ of agricultural land loss. To offset the loss of agricultural land, Northeast China Plain and Xinjiang were reclaimed. Substantial recovery of forests (49,908 km^2^) and closed shrubland (60,854 km^2^) occurred in mountainous regions due to abandoned infertile farmland, secondary succession, and governmental conservation policies. The spatial patterns and trends of LCLUC in Northern China provide information to support effective environmental policies towards sustainable development.

Since the Industrial Revolution, the growing pace and intensity of human activities have profoundly accelerated the land cover/land use changes (LCLUC)^1^,^2^. As a major driver of climatic and environmental changes from local to global scales^3^,^4^, LCLUC has received global concerns and become an important challenge in the studies of coupled human and nature systems^5^,^6^.

LCLUC alters landscape structure^7^, surface energy balance^8^, soil properties^9^, and water and nutrient cycles^10^,^11^, and thus impacts ecosystem services^12^ and climate^8^,^13^. LCLUC also raises the vulnerability of humans to natural disasters^14^. For example, the human settlements close to forest interiors make the populations vulnerable to the wildfire hazards^15^. In addition, economic globalization induces tight connection of LCLUC around the world: Rapid forest recovery in wealthy countries is often associated with massive deforestation in others via world trade^16^.

Monitoring LCLUC and exploring their spatial pattern dynamics have drawn much attention recently^6^. Remote sensing imagery has been used to assess the LCLUC due to its high consistency across spatiotemporal scales^17^. Early LCLUC studies were based on imagery of low spatial resolution at 1 km or above, but it is common today to use resolution at 30 m or less^18^. Based on the land cover derived from Landsat images, Etter *et al*. identified hotspots of deforestation and reforestation at local scale during a time period by subtracting the forest cover of the starting year from that of the ending year^19^. Since land cover recognition based on spectral reflectance of remote sensor is strongly affected by extreme climate events such as El Niño, land change detection by Etter *et al*.’s methods may be prone to extreme climate events at the starting or ending year. To compensate, the trajectory-based change detection was proposed to identify land changes between forest and non-forest using Normalized Difference Vegetation Index (NDVI) time series^20^. Furthermore, significant land change hotspots were detected by means of linear regression to fit slopes of land cover time series at the municipality scale^21^. However, the spatial heterogeneity of land changes within municipalities is ignored, and the temporal slope of land cover change strongly depends on the size of municipalities which could bias the associated spatial pattern of land change in a region. Therefore, it is critical to conduct land change trend analyses at fine and consistent scale in order to capture the hotspots and to reveal the underlying mechanisms.

China has been under unprecedented economic growth, massive population migration, and enormous land change over the past decades^22^,^23^. The fast-growing economy gave rise to the off-farm employment opportunities in cities and towns^24^, resulting in changes in demographic structure and composition across China^25^,^26^. Urban expansion, especially in the coastal and provincial cities, increased pressures on limited land resources in suburban and incurred loss of vegetation for impervious surface^27^,^28^. At the same time, the industrialization accelerated the marginalization of the inferior farmland especially in central west China due to the rising costs of farming^29^. Furthermore, growing demand for forest products enhanced the competition between forestry and agriculture^24^. The economy pushed the farmland in less favorable areas (e.g., steep slope areas) out of agricultural production.

Recognizing the consequences of environmental degradation and crisis, the Chinese government implemented a number of ecological restoration policies to promote the conversion of inappropriate farmland to grasslands and forests^30^. For instance, the Natural Forest Conservation Program (NFCP) and the Grain for Green Program (GFGP) fund the farmers for restoring ecosystem services^31^. Specifically, the NFCP protects and conserves natural forest by closing mountains, banning commercial logging, and providing incentives for afforestation. GFGP subsidizes farmers to convert marginal farmland to forest or grassland. Under the main trend of agricultural abandonment, reclamation of new farmland occurred in areas suitable for machine farming. The above processes and activities profoundly altered the spatial pattern of landscapes. However, there is limited knowledge about the spatial distribution of the rates and magnitudes of LCLUC in contemporary China. It is important to develop a land change detection method to explore spatial patterns of LCLUC and to quantify the rates and magnitudes of land change.

In this study, we mapped the yearly land cover of Northern China for 2001–2013, and proposed a method to detect land change hotspots by computing the temporal slope and magnitude of land cover changes. We explored the land change spatial patterns and tested the following hypotheses to answer the question of “where” and “how” LCLUC were occurring in Northern China: (1) urban expansion has pressured limited land resources around the coastal and provincial cities with the loss of fertile arable land, (2) new farmland has been reclaimed to meet the needs, and (3) forest recovery has occurred around the rural mountain areas due to the polices of conservation and the abandonment of farmland.

## Methods

### Study area

**Figure 1:**
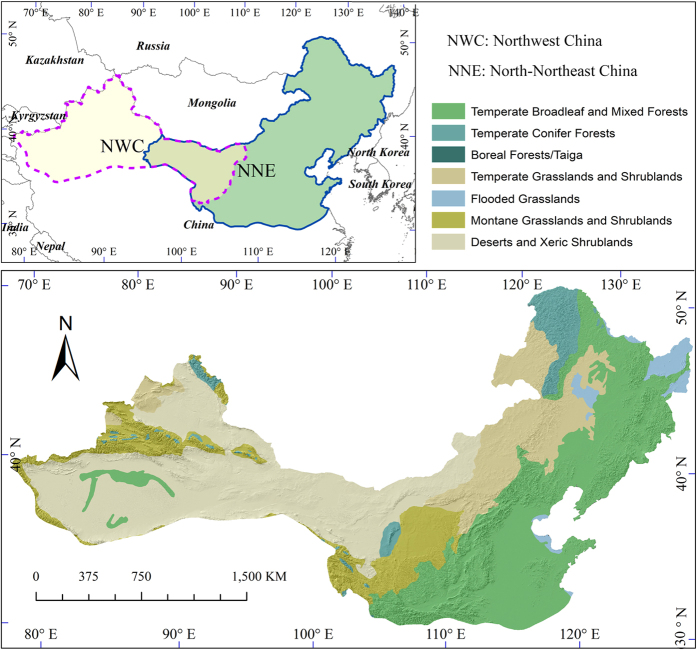
Study Area of Northern China. The upper left panel shows two sub-regions for land cover classification purpose: northwest China (in pale orange) and north-northeast China (in light green) with an overlay in the middle (in lime green). The bottom panel presents the 7 biomes distributed in northern China (Data sources: The Nature Conservancy, 2012. tnc_terr_ecoregions: vector digital data available at *http://maps.tnc.org/gis_data.html; Metadata available at http://maps.tnc.org/files/metadata/TerrEcos.xml*). Map created using ArcGIS 10.0 (Esri, CA, www.esri.com).

The Northern China (NC), geographically ranging over 32.47°–53.55°N and 73.84°–134.77°E and varying from 0 to 4,911 m a.s.l. in elevation (Fig. 1), stretches from the humid monsoon region in the east to the arid continental region in the west, and features great longitudinal rainfall gradient and diverse zonal vegetation types. The southwest of NC is bounded by the Qinghai-Tibet Plateau. NC covers most transitional area among the three major natural zones of China, i.e., the eastern monsoon zone, the northwestern arid and semi-arid zone, and the Qinghai-Tibet alpine zone. With the total area of 4,566,804 km^2^, NC supports approximately 5.8 billion people in 1,300 counties belonging to 16 provinces at the year of 2010^32^. Ecological restoration policies have been implemented since 1998 to control land degradation and soil erosion, and to protect the natural forests^17^. Governmental programs in this region have encouraged the conversion of existing inferior agricultural land to forest, scrubland, or grassland.

### LCLU Mapping

The LCLU classification methodology in this study is generally outlined by Wang *et al*.^17^ with modifications to fit the study at subcontinental scale. One important step of classification is to obtain the ground reference data in order to train the classifiers and to validate the classification. According to the visual interpretation schema in the previous studies^17^,^33^,^34^ and relevant literatures^35^,^36^, we intend to classify land cover into deciduous broadleaved forest (DBLE), deciduous needle-leaved forest (DNLE), evergreen broadleaved forest (EBLE), evergreen needle-leaved forest (ENLE), mixed forest (MIXED), closed shrubland (CLSH), open shrubland (OPSH), grassland (GRAS), sparse vegetation (SPAS), urban area (URBN), agricultural land (AGRI), bare ground (BARE), permanent snow cover (SNOW), and water (WATR). In this study, a total of 14,909 ground reference points were collected by means of a similar approach used in our study on the Agro-Pastoral Transition Band in Northern China^17^. The ground reference points were identified in a semi-random manner with the following criteria to minimize the spatial autocorrelation and the effects of triangular PSF (point spread function) along the scan direction^37^ of MODIS sensor: 1) the distance between any pair pixels is greater than 1,500 m, and 2) each reference point as a MODIS pixel is located within a uniform land cover patch on Google Earth images. The process of reference point identification was aided by the historical vegetation maps^38^, the high resolution images and user-pinned photos on Google Earth (GE), and the species distribution information from Flora of China (http://www.eflora.cn/). We listed the typical remote sensing images, corresponding ground photographs (available at Google Earth), and brief description of locations for the LCLU types within Northern China in a table (Supplementary Table S1).

Seasonal MODIS reflectance of blue, red, near infrared and mid infrared bands and vegetation indices (VIs), as well as the phenological parameters derived from the MODIS EVI (Enhanced Vegetation Index) product (MOD13Q1, 16-Day Level 3 Global 250 m SIN Grid, Collection 5)^39^, were used as variables to classify LCLU for each calendar year from 2001 to 2013. In addition, topographic features, such as elevation, slope, and aspect, were included to complement the remote sensing data due to their important roles in vegetation distribution.

Due to the great difference in environments, we divided our study area into north-northeast (NNE) and northwest (NWC) sub-regions with overlaysas as illustrated in upper left of Fig. 1, and then trained and tested the classifier for each sub-region separately. Specifically, for each sub-region, we extracted the abovementioned variables for the ground reference points and randomly split the points into two subsets: 70% for training and the remaining for testing. For the training subset, we formed three combinations of predicates as the training inputs: C1 includes the growing-season statistics of MODIS reflectance and VIs, the phenology parameters, and the topographical features; C2 is a modification of C1 by substituting four-season statistics of VIs for the phenology parameters; and C3 encompasses all the variables in C1 and C2. Random forest (RF) classifier was used in this study because RF was proved to be more robust and efficient than other classifiers^17^. RF was trained and tested by each of the three datasets to obtain three classification models for each sub-region. We quantified classification accuracy using overall classification accuracy (OA), producer’s accuracy (PA), user’s accuracy (UA), and Kappa statistics. For each sub-region, we applied the three models separately and derived the LULC classification maps as well as the corresponding class membership probability datasets at pixel level. If the classification was consistent across three models, the final classification for the pixel was set to the agreed LCLU type (occurred for most of the pixels). If not consistent, Bayesian-average integration was applied to set the final LCLU type according to the classified type and the corresponding membership probability of each model. This approach was proved to yield high accuracy in LCLU mapping^17^. The classification results for the two sub-regions were finally mosaicked to obtain the LCLU maps for the whole Northern China.

### Trend Analyses to identify LCLUC hotspots

**Figure 2:**
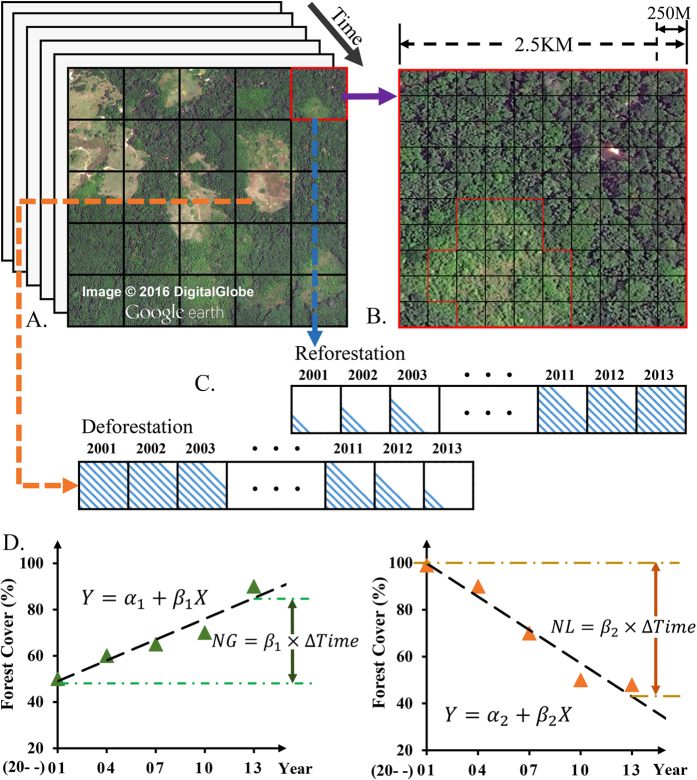
Illustration of land changes in the big grid and linear regression for each land cover type (e.g. forest land change). Land cover in a series of big grids (**A**); Each big grid cell contains 10 × 10 land cover pixels (**B**); Forest cover dynamics as a time series in one big grid cell (**C**); Regression of forest cover on time (**D**). NG, net gain, NL, net loss.

Hotspot analyses focused on the land conversions among AGRI, BARE, CLSH, FORE (including all forest types), GRAS, OPSH, SPAS, and URBN. Hotspots of land change were identified based on the rate of change over the 13 years. We modified the LCLUC hotspots detection used in LCLUC for the Latin America and the Caribbean regions^21^,^40^ by linear regression for each big grid cell. The approach intended to catch spatial heterogeneity of LCLUC at local scales and to provide unbiased mapping of temporal trend of LCLUC. Specifically, we first calculated the fractions of the eight land cover types in each big grid cell of 2.5 × 2.5 km^2^ (10 × 10 MODIS pixels, Fig. 2). The size of the big grid cell was proved to minimize the ‘speckle’ in land cover maps and to capture the trend of LCLUC^19^. This allowed us to obtain a time series of annual land cover fractions for each big grid cell. Simple linear regression of the land cover fraction series on years enabled us to obtain a trend slope with associated significance (*p*-values). Gain or loss of each land cover type was computed as the significant slope (*p*-value < 0.1) multiplied by the time interval of 13 years (Fig. 2). We also estimated the uncertainties of the net land change by multiplying the standard error of the slope by 13 to give the one standard deviation of the net land cover change, SD_*LCC*_.

We further analyzed the land change hotspot at large scales by overlaying the land change raster with a biome map to quantify the overall rates of change at different biomes. Spatial patterns of urbanization, agriculture abandonment and reclamation, and reforestation were explored to reveal the interrelationships. In specific, relationship between urbanization and farmland displacement was obtained by calculating areas of net land transfer within Jing-Jin-Ji Urban Agglomeration, one of the three largest urban agglomerations in China. We also illustrated the land changes between agriculture and grassland, and the shift from shrubland to forest. Specifically, we quantified the woodland (including shrubland and forest) dynamics in three montane areas: Lüliang Mountains, Taihang Mountains, and Yan Mountains.

## Results

### Accuracy of LCLU mapping

Among the three input datasets, the highest overall accuracies and Kappa statistics were achieved for the predicator combination 3 which included the reflectance, VIs, phenology, and terrain information for both NNE and NWC sub-regions (Table 1). The average overall accuracies were 85.1±0.6% and 87.2±1.2% for NNE and NWC, respectively. Classification accuracies were higher for AGRI, BARE, EBLE, SNOW, URBN, and WATR than for other land cover types. The forest covers in NWC had relatively high accuracies with average PA of 91.5% and UA of 95.4%, due to the strong contrast between forest and desert vegetation in NWC. Specifically, DNLE had greater than 90% accuracies for C2 and C3 in NWC, whereas only less than 70% in NNE since DNLE in NNE was likely to be mixed with DBLE. PA for MIXED was relatively lower (62~67%) than those of other land cover types.

### Trend of land cover changes

**Figure 3:**
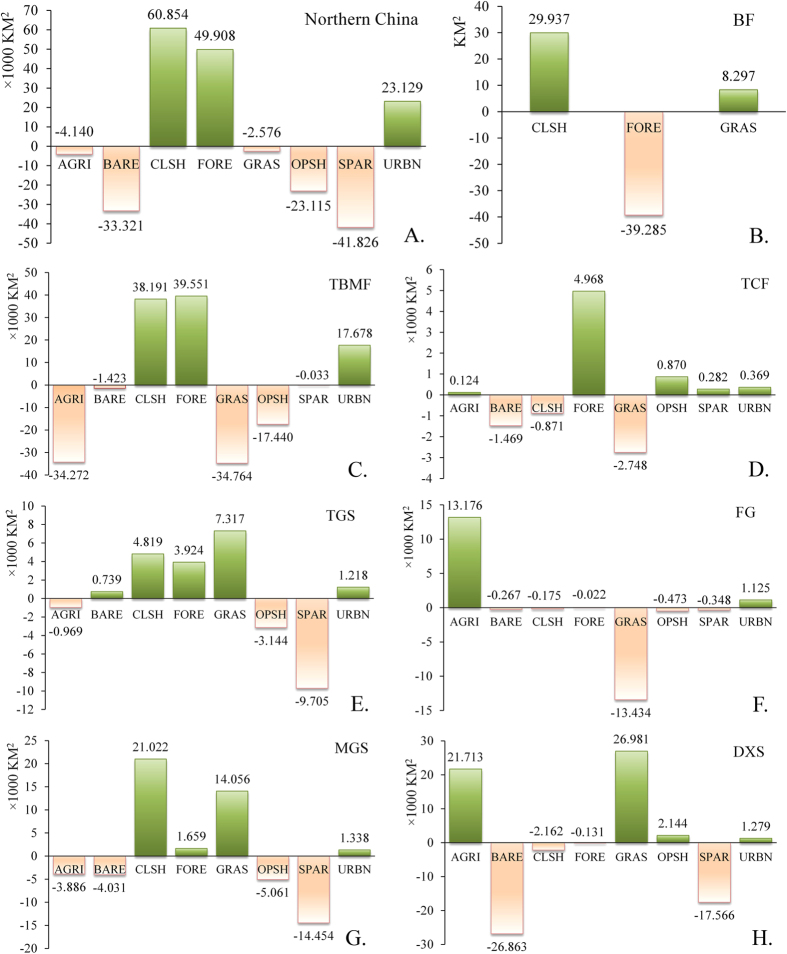
Land cover net gain and net loss in Northern China (NC, panel A) and in the seven biomes in NC (panels B–H. (**B**) Boreal Forests, (**C**) Temperate Broadleaf and Mixed Forests, (**D**) Temperate Conifer Forests, (**E**) Temperate Grasslands and Shrublands, (**F**) Flooded Grasslands, (**G**) Montane Grasslands and Shrublands, (**H**) Deserts and Xeric Shrublands). Map created using ArcGIS 10.0 (Esri, CA, www.esri.com).

Forest and closed shrubland in Northern China (Fig. 3A) increased substantially during 2001–2013 period with a net gain of 49,908 km^2^ (8.5% of 2001) and 60,854 km^2^ (20.5%), respectively. Likewise, urban expanded with a net gain of 23,129 km^2^ (91.7%). Net gain of these land cover types costed massive declines of bare ground, open shrubland, and sparse vegetation summed to a total of 98,262 km^2^ (−5.1%). Agriculture and grassland also lost 4,140 km^2^ (−0.5%) and 2,576 km^2^ (−0.3%), respectively.

Land cover change differs among biomes (Fig. 3B–H). Although urban expansion was observed in all biomes except boreal forests (BF), approximately 76% of urban gain was concentrated in the temperate broadleaf and mixed forests biome (TBMF). Agriculture declined greatly in TBMF (−34.3 × 10^3^ km^2^), but gained in the flooded grasslands biome (FG, or wetland) (13.2 × 10^3^ km^2^) and the deserts and xeric shrublands biome (DXS) (21.7 × 10^3^ km^2^), which incurred the loss of grassland in FG, and the loss of bare ground and sparse vegetation in DXS. The forest cover increased in all biomes except BF, FG, and DXS where forest cover encountered slight decrease. Forest gain ranged from 1,659 km^2^ in the montane grasslands and shrublands biome (MGS) to 39,551 km^2^ in the TBMF biome. The closed shrubland increased, but the open shrubland decreased in the biomes of TBMF, temperate grasslands and shrublands (TGS), and MGS. The sparse vegetation decreased greatly in the biomes of TGS, MGS, and DXS.

### Spatial patterns of land cover change hotspots

#### Urbanization and Agriculture Conversion

**Figure 4:**
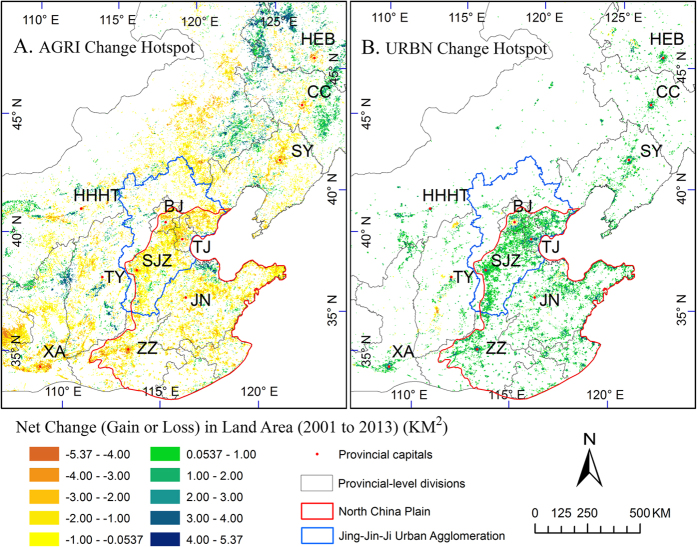
Net changes of agriculture (**A**, AGRI) and urban area (**B**, URBN) from 2001 to 2013 in a part of north-northeast China. The land change is significant for *p*-value < 0.1. BJ, Beijing, TJ, Tianjing, and SJZ, Shijiazhuang. Map created using ArcGIS 10.0 (Esri, CA, www.esri.com).

The spatial patterns of land cover change (Fig. 4) indicated that the significant loss of agricultural land in North China Plain (with the boundary in red) during 2001–2013 was associated with urban expansion around the provincial capital cities. This association was especially strong for the Jing-Jin-Ji (Beijing, Tianjin, Hebei) Urban Agglomeration (as labeled with blue boundary) which pioneered urbanization in China. We calculated net changes of the two land cover types within the Jing-Jin-Ji Urban Agglomeration. The results showed a tremendous urban expansion of 6,387 km^2^, but a massive loss of 6,601 km^2^ of fertile agricultural land in the Urban Agglomeration. The cities of Tianjin, Tangshan, Beijing, and Shijiazhuang had high urban expansion speed during 2001–2013 with the net gain of 1,183, 1,062, 700, and 638 km^2^, respectively.

#### Agricultural Reclamation

**Figure 5:**
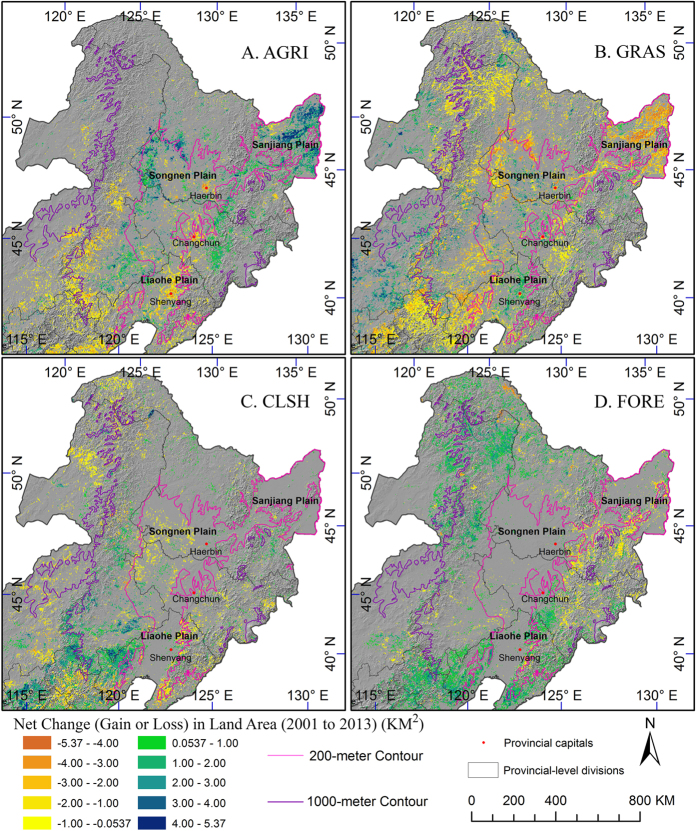
Net changes of agriculture (**A**), grassland (**B**), closed shrubland (**C**), and forest (**D**) from 2001 to 2013 in northeast China. Changes are significant with *p*-value < 0.1. Map created using ArcGIS 10.0 (Esri, CA, www.esri.com).

In contrast to loss of agricultural land around cities, new agricultural lands were also reclaimed to sustain food production in northeast China. Areas with significant agriculture gain (Fig. 5A) approximately matched those with grassland loss (Fig. 5B). The new agricultural lands were mainly located in the northeastern Sanjiang Plain, the north and southeast edge and west of Songnen Plain, and the northwestern Liaohe Plain (Fig. 5A). The net gain of agricultural land and net loss of grassland in Sanjiang Plain were 12,523 and 12,399 km^2^, respectively, thus almost all these new agricultural reclamations were in the price of grassland loss. Contrary to the gain in the plains and lowland, agriculture in upland (around the 1,000-meter contour in the bottom left of Fig. 5A) declined and was converted to grassland, closed shrubland, or forest.

**Figure 6:**
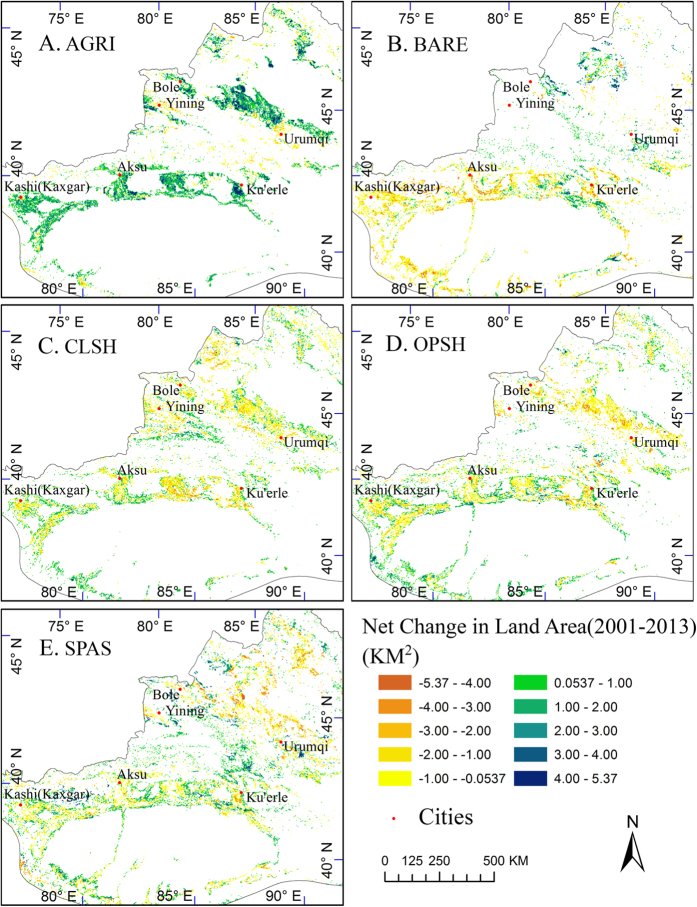
Net Changes of agriculture (**A**), grassland (**B**), closed shrubland (**C**), open shrubland (**D**), and sparse vegetation (**E**) from 2001 to 2013 in the northwestern China. Land changes are significant with *p*-value < 0.1. Map created using ArcGIS 10.0 (Esri, CA, www.esri.com).

Significant agricultural land expansion (Fig. 6A) was also detected in Xinjiang province with arid environment. The comparison of the agriculture gain in Fig. 6A to the land cover changes in Fig. 6B–E revealed that the gain in agriculture was associated with the loss in shrubland, sparse vegetation, and bare ground. The total agricultural land gain of 25,418 km^2^ in Xinjiang mainly came from the previous closed shrubland (−4,527), open shrubland (−5,797), sparse vegetation (−6,421), and bare ground (−5,840).

#### Woodland Recovery

**Figure 7:**
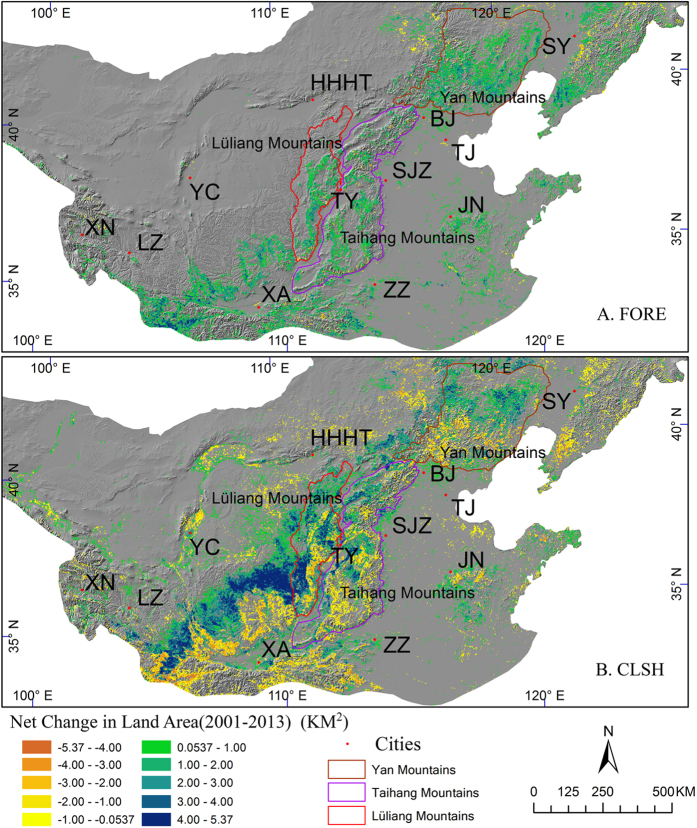
Gain and loss in: Forest (**A**) and Closed Shrubland (**B**) from 2001 to 2013. Land changes are significant with *p*-value < 0.1. Map created using ArcGIS 10.0 (Esri, CA, www.esri.com).

The spatial patterns of net forest change for 2001–2013 (Fig. 7A) showed regional heterogeneity. Forest gains were mainly occurring in the mountains. A comparison between Fig. 7A and B revealed forest recovery advanced into the previous closed shrubland. Forest gains in Lüliang Mountains and Taihang Mountains reached 2,611 and 3,789 km^2^, in the price of declined closed shrubland (the major contributor, Fig. 7B) of 2,093 and 3,437 km^2^, respectively. However, the woodland dynamics in Yan Mountains was different: Forest gained in the south and middle of the mountains by 8,531 km^2^ which incurred the loss of 3,925 km^2^ of closed shrubland mostly in the south, but closed shrubland gained in the central mountains.

## Discussion

### Methodology and Uncertainty

We adopted and modified a robust LCLU classification method to incorporate multiple classification models and posterior data fusion^17^. This LCLU mapping was based on a reliable ground reference data collection strategy and could yield high statistical accuracy, and provided pixel-level uncertainties which were essential for subsequent analyses and applications. We implemented a hotspot detection method of LCLUC at big grid cells, rather than at municipalities. The hotspot detection in previous studies^21^,^40^ at municipality level ignored the spatial heterogeneity within municipalities and could bias the land change trend and associated spatial pattern due to the inconsistent municipality size. Slope and change based on linear regression are less prone to the error caused by extreme climate events than the approach to take the difference between the beginning and ending years. Furthermore, the standard error of the regressed slope allowed us to assess the significance and uncertainty in the land cover changes at big grid level. The modified LCLU mapping and the improved hotspot detection contributed to the mechanistic understanding of LCLUC dynamics and pertinent socioecological consequences at regional to global scales. Policy makers could also be benefited to propose rational land use policies that balance between human demands and environment protection for sustainable use of land resources.

There exist limitations with the proposed LCLUC hotspot detection. We could not detect the changes at scales less than 5.3 ha due to the medium spatial resolution of MODIS images. Another limitation is that the linear regression is unable to catch the nonlinear LCLUC caused by severe disturbances. In the assessment of uncertainties of forest change, we showed (Supplementary Figure S2) that 87.7% of the big grid cells had their estimated errors of net forest change lower than 1 km^2^. This is the case for mountainous area such as Taihang and Lüliang Mountains where forests spontaneously regenerated so that the estimated errors were relatively low (Supplementary Figure S2). The estimated errors could be large when there were disturbances such as fire (Sites A, B, C in Fig. S2). For example, site A encountered a fire disturbance in 2003, spread from the grassland in the west, and thus showed a high error^41^.

### Urbanization and Agricultural Lands Replacement

Other than urban sprawl in most developed countries (e.g., US)^42^,^43^ or excessive urbanization in the developing countries in Latin America^44^, urbanization in China is characterized by rapid and excessive land conversion which is often ahead of urban population growth^45^,^46^. According to our result, urban lands expanded significantly with associated neighborhood agricultural land decline during 2001–2013 in Northern China, especially in the Plains (e.g. North China Plain and Northeast China Plain, Fig. 4A and B), which concurred with existing studies^28^,^47^. The net urban gain of 23,129 km^2^, an increase of 91.7%, is also comparable with the report in the literature^48^. Neighborhood farmlands replacement by urban expansion could be attributed to the rapid growing economy in China, which leads to a rise in both the off-farm employment opportunities and the income of secondary and service sectors. The trend of economy is especially strong in the coastal and provincial cities (red dots in Fig. 4), and has incurred rural population to surge continuously into cities^23^,^49^. In addition, the much higher land price in cities than in rural areas stimulates the advance of cities towards rural neighborhoods, which swallows up fertile farmland but boosts the governments’ revenues^28^,^50^. The incomes from land conversion, as high as 30–70% of municipal revenue in many cities, are invested in the facilities and infrastructures of the cities^28^,^51^ which in turn creates jobs and leads to further urban expansion. Facing risks of food security and urbanization foam, the Chinese government has implemented policies and laws aiming at halting the loss of farmland and regulating the real estate market^46^,^49^. The effect of agricultural protection policies on land conversion is not imminent, however, the spatial growth of urban area has slowed down^49^.

### Agricultural Lands Abandonment and Reclamation

Global agriculture towards mechanization and intensification^43^,^52^ is accompanied with abandonment of marginal farmlands^53^,^54^. In addition to the neighborhood farmlands loss due to urbanization, our results revealed high spatial heterogeneity in agricultural land change patterns across Northern China. The agricultural land decreased in the eastern and central mountain regions, merely changed in those basic farmland protection areas set up by the central government in the Plains, but significantly increased in the flat regions of the northeastern (e.g., Heilongjiang province, Fig. 5A) and the northwestern (e.g., Xinjiang province, Fig. 6A) China. The newly reclaimed agricultural land offsets the loss induced by urban expansion and agricultural abandonment, resulting in no significant increasing or decreasing trends for the total area of farmland in Northern China (*p*-value = 0.7).

In general, agricultural abandonment can be ascribed to the facts that: the remote smallholders were outcompeted by the large enterprises due to lack of access to markets, investment, and new farming technology^55^, and the economically attractive off-farm jobs pulled the labors out of farming^24^,^55^. In China, in addition to displacing farmland (Fig. 4), the growing economy and associated urbanization promote the labor costs and the rural-to-urban migration, and thus accelerate the marginalization of the inferior farmland^29^,^47^. Long-term irrational cultivation at steep slopes causes soil erosions and fertility degradation, and some degraded farmlands were therefore abandoned to give way to natural recovery^56^. Many ecological restoration policies also fund the farmers to convert the inferior or marginal farmland to forest or grassland. Farmland abandonment in China could be attributed to economic development, rural-urban-migration, land degradation, and ecological restoration policies and programs.

The rising food demands for a growing population have stimulated the reclamation of new farmland suitable for machine farming. Our results are consistent with the reports that farmland (i.e., paddy rice) in the eastern Sanjiang Plain expanded significantly due to the conversion of large flat grasslands (e.g., marshes) since late 1980 s^22^,^57^,^58^. Technology advances make it possible to expand farming in dryland of Spain by large farming enterprises to maximize the use of dry and sunny areas with high productivity potentials^55^. In this study, we found a similar phenomenon that the farmland reclamation and the state farms sector reached high mechanized level in the arid and semi-arid region of Xinjiang province (Fig. 6A), where the drylands were previously only suitable for grazing. Yi *et al*.^58^ reported that more than 3,725.93 km^2^ unused land were converted to farmlands in Xinjiang during the period of 2000–2010, which concurred with our result that the farmlands had largely expanded into bare grounds, sparse vegetation lands, and shrublands (Fig. 6B,C,D and E).

### Woodland Change Dynamics

Global forest cover undergoes continuous decline, whereas forest changes present spatiotemporal heterogeneity among countries. Deforestation has been dominant in tropical regions since 1980 s^1^,^59^. However, reforestation has emerged in many temperate countries and also some tropical areas after a long-term decline, such as European countries^60^,^61^, United States^62^,^63^, India^64^, and Puerto Rico^7^. Forest Transition theory was proposed to understand the mechanisms of forest dynamics shifting from deforestation to reforestation in a country^65^. Economic growth and forest scarcity are highlighted in the Forest Transition theory, which ascribed forest recovery to the rural-to-urban migration and the rising farming costs^24^.

China has experienced extensive forest recovery since 1980s^22^,^66^. Huge afforestation programs and forest protection policies are believed to take important roles in this process^17^,^31^. Our results supported that afforestation and reforestation prevailed in Northern China with significant woodland expansion around the mountains over the period of 2001–2013 (Figs 3, 5 and 7). The increasing trend of the total area of woodland in Northern China is significant at *p* < 0.001. Forests and closed shrublands gained in this period (Fig. 3) by 49,908 km^2^ or 8.5% and 60,854 km^2^ or 20.5%, respectively. These substantial increases in woodland could be a result of the massive rural-to-urban migration, the abandoned marginal farming in low-hilly regions, and the governmental subsidy to farmers for conservation^67^. The abandoned farmlands were either planted with trees or gradually replaced by spontaneous growth of grass, shrubs, and trees via secondary succession^17^. For instance, in most regions of Lüliang Mountains and Taihang Mountains and the southern region of Yan Mountains, the forest gains were achieved primarily from the previous closed shrubland (Fig. 7). Existing remnant trees in forest patches in the mountains not only serve as seed sources but also attract animal dispersers^68^, and thus could accelerate spontaneous regeneration of secondary forest. Technically, the planted saplings are likely to be classified initially as shrubland by remote sensing but later as forest when the trees grow up. In the middle region of Yan Mountains, both the closed shrublands and the forests expanded rapidly. It implies that the woodland expansion might be accomplished not only by spontaneous regeneration via succession but also by afforestation due to programs and policies, such as BTSSCP (Beijing and Tianjin Sandstorm Source Control Project) and GFGP implemented in this region (Supplementary Figure S1). In addition, recent increasing trend of rainfall in Northern China might also favor the regrowth of woodlands (unpublished data). This conclusion was supported by others who reported vegetation increased over the most BTSSCP region during 2000–2010^69^. The woodland recovery in mountainous areas proves the effectiveness of the ecological restoration policies and could guide future policy making.

## Additional Information

**How to cite this article**: Wang, C. *et al*. Spatially differentiated trends in urbanization, agricultural land abandonment and reclamation, and woodland recovery in Northern China. *Sci. Rep.* **6**, 37658; doi: 10.1038/srep37658 (2016).

**Publisher’s note:** Springer Nature remains neutral with regard to jurisdictional claims in published maps and institutional affiliations.

## Supplementary Material

Supplementary Information

## Figures and Tables

**Table 1:** Classification accuracies for two sub-regions with three input datasets (NNE: north-northeast; NWC: northwest).

	Producer’s Accuracy (%)						User’s Accuracy (%)					
	NNE (training: 9,881)			NWC (training: 8,927)			NNE			NWC		
	C1	C2	C3	C1	C2	C3	C1	C2	C3	C1	C2	C3
AGRI	94.0	94.4	94.4	91.3	91.6	91.9	93.7	94.4	94.9	91.0	91.0	92.2
BARE	86.8	87.4	86.3	92.6	94.0	93.3	94.0	97.0	96.9	96.7	97.8	97.8
CLSH	78.4	78.8	80.8	78.9	80.2	81.0	82.0	82.7	83.4	78.9	78.8	80.7
DBLE	88.6	87.5	89.0	90.3	90.3	88.7	73.7	74.8	73.8	98.2	100	100
DNLE	62.9	67.4	65.2	79.7	91.6	93.0	67.5	69.8	69.9	95.0	95.6	96.4
EBLE	88.3	88.3	88.3	—	—	—	93.2	90.7	91.9	—	—	—
ENLE	80.7	79.4	81.2	96.7	96.7	96.7	87.8	86.8	88.3	88.2	92.8	92.8
MIXED	62.4	66.9	65.4	—	—	—	80.6	80.9	82.9	—	—	—
GRAS	84.4	87.0	86.2	84.1	85.7	84.8	82.5	85.2	84.7	82.9	86.1	84.4
OPSH	74.2	77.3	77.3	79.2	81.2	81.6	75.0	75.7	75.7	70.7	71.0	71.6
SNOW	100	100	100	100	100	100	96.9	96.9	96.9	97.9	97.9	98.3
SPAS	83.1	85.1	86.2	74.6	76.3	76.3	71.7	73.1	72.7	79.9	84.2	83.4
URBN	90.5	89.0	88.0	91.6	91.6	92.9	93.8	93.7	94.1	94.0	93.4	92.9
WATR	93.9	94.6	93.9	93.9	93.0	93.9	100	100	100	99.1	100	100
***OA***	84.5	85.4	85.5	86.0	87.7	87.8						
***Kappa***	82.8	83.9	84.0	87.4	88.9	89.0						
