# Supplementary material for: Spatially differentiated trends in urbanization, agricultural land abandonment and reclamation, and woodland recovery in Northern China
Source: Sci Rep. 2016 Nov 22;6:37658. doi: 10.1038/srep37658 (PMC5118683; doi:10.1038/srep37658)
Supplement: Supplementary Information [file srep37658-s1.pdf]

Spatially differentiated trends in urbanization, agricultural land abandonment and reclamation, and woodland recovery in Northern China

Chao Wang, Qiong Gao, Xian Wang, and Mei Yu*

Department of Environmental Sciences, University of Puerto Rico, Rio Piedras

San Juan, PR 00936, USA * Email: meiyu@ites.upr.edu; Tel: 1-787-764-0000 x 4375

Supplementary Figure S1

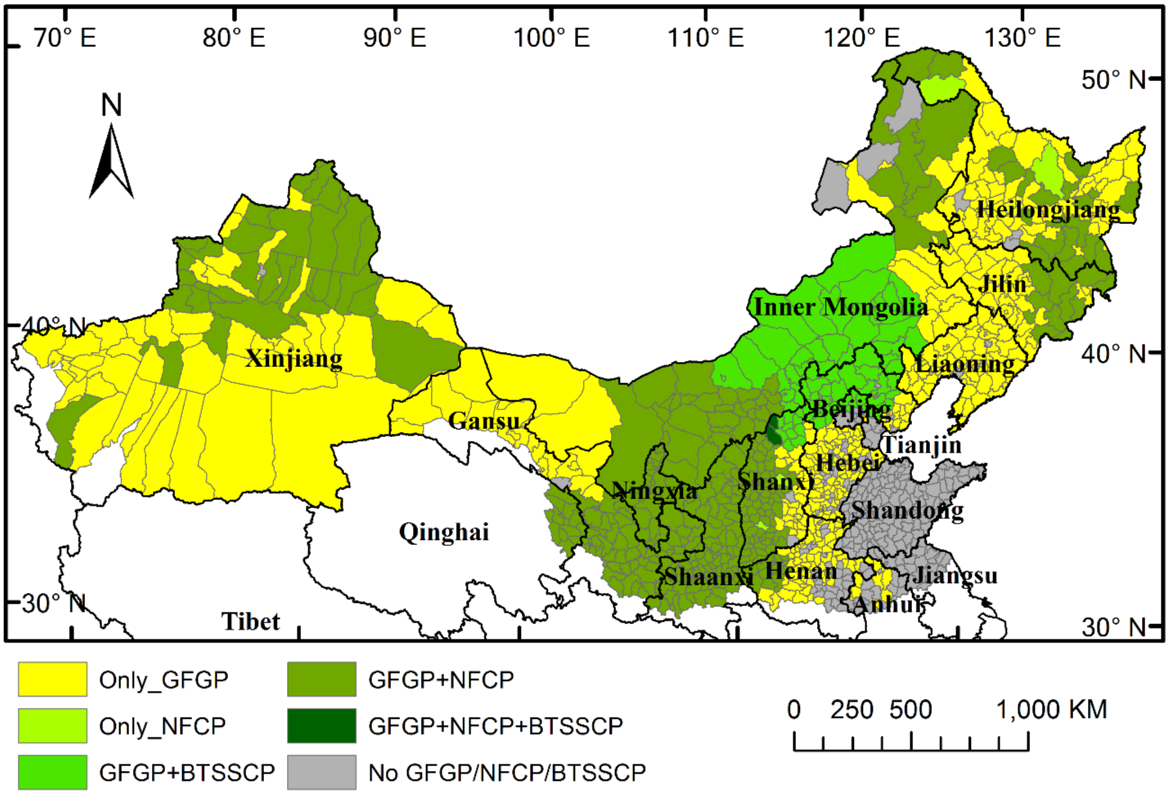

Figure S1 Three main ecological restoration programs implemented by Chinese government in northern China since 1998. GFGP, Grain for Green Program, NFCP, Natural Forest Conservation Program, and BTSSCP, Beijing and Tianjin Sandstorm Source Control Project. Map created using ArcGIS 10.0 (Esri, CA, www.esri.com).

Supplementary Figure S2

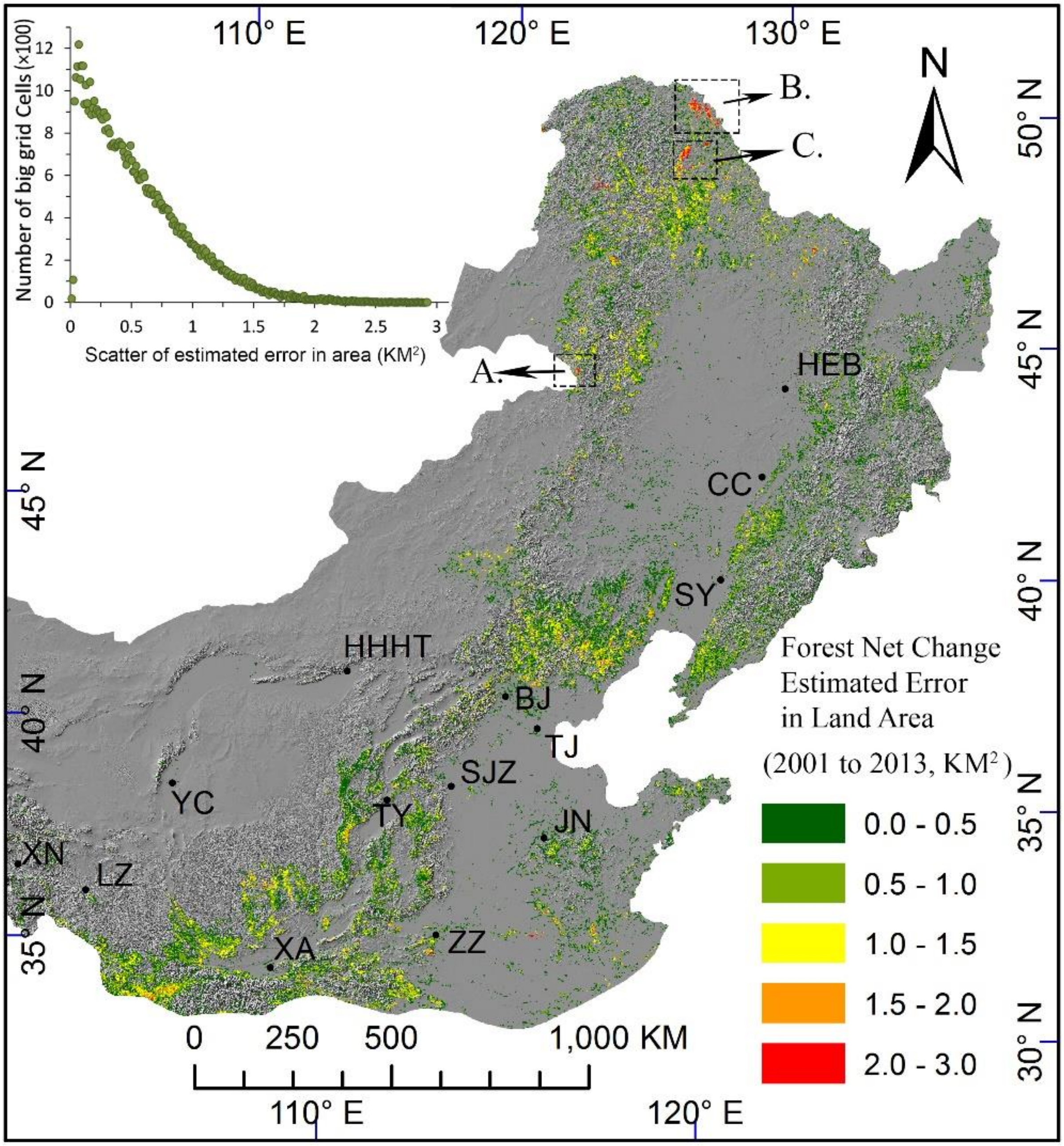

Figure S2 Estimated error of net forest change and its statistical distribution from 2001 to 2013 in a part of north-northeast China. Map created using ArcGIS 10.0 (Esri, CA, www.esri.com) and Microsoft Office Excel 2013.

Supplementary Table S1

Table S1 Landscape photographs and descriptions of land cover types (photographs and images are from Google Earth Pro). AGRI, agricultural land; BARE, bare ground; CLSH, closed shrubland; DBLE, deciduous broadleaved forest; DNLE, deciduous needle-leaved forest; EBLE, evergreen broadleaved forest; ENLE, evergreen needle-leaved forest; GRAS, grassland; OPSH, open shrubland; URBN, urban area; and WATR, water.

Class	Physical photographs in GE	Images in GE	Description
AGRI	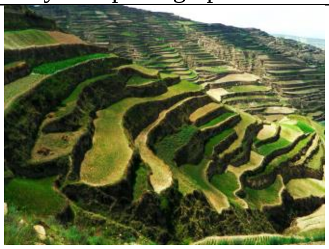	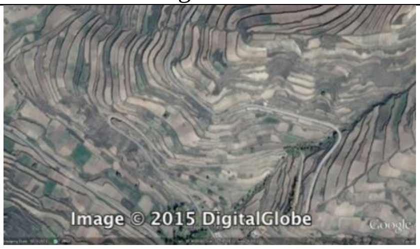	Located in Lanzhou, Gansu (35.959°N, 103.650°E)<br>It has rectilinear shapes on the whole mountains.<br>Photograph was taken on 2009/06/25.<br>The acquisition date of this imagery was 2013/10/10.
BARE	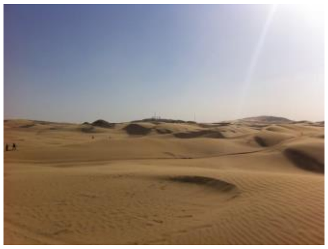	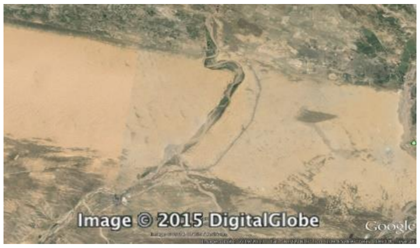	Located in Ordos, Inner Mongolia (40.217°N, 109.904°E)<br>It has yellow color, the texture of sand dune.<br>Photograph was taken on 2011/04/26.<br>The acquisition date of this imagery was 2013/04/10.
CLSH	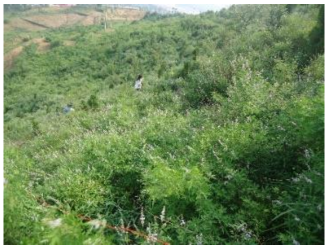	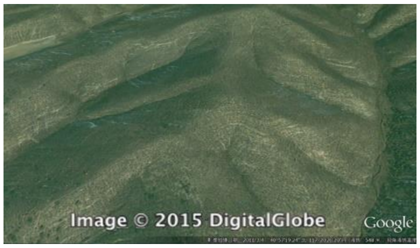	Located in Chengde, Hebei (40.616°N, 110.703°E)<br>It has dark green color, texture of mountain and more dots compare to open shrub.<br>Photograph was taken on 2010/07/29.<br>The acquisition date of this imagery was 2011/03/04.
DBLE	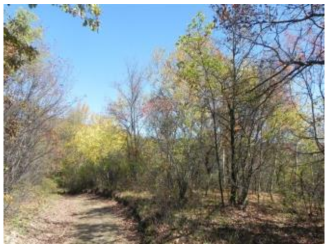	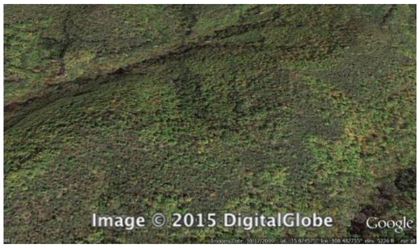	Located in Qingyang, Gansu (35.868°N, 108.494°E)<br>It has green and yellow color, texture of mountain and hairy objects which are trees in the mid of Oct., when some leaves begin turning yellow.<br>Photograph was taken on 2011/10/01.<br>The acquisition date of this imagery was 2009/10/17.
DNLE	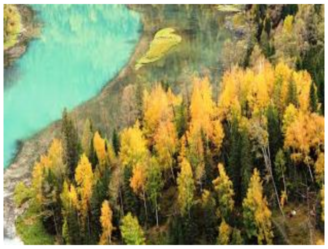	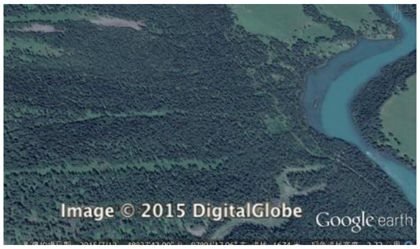	Located in Altay, Xinjiang (48.626°N, 87.033°E)<br>It has dark green and rough canopy. We identify if the forest is coniferous by the physical photograph and locations of the dominated species in China.<br>Photograph was taken on 2013/09/12.<br>The acquisition date of this imagery was 2015/07/12.

<table>
<tr><td>EBLE</td><td>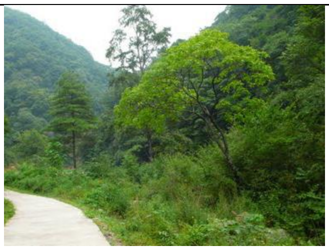</td><td>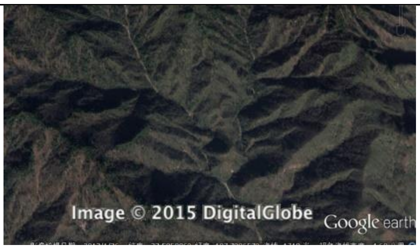
</td><td>Located in Hanzhong, Shannxi
(33.620°N, 107.807°E)
It has smooth canopy. We identify if the forest is evergreen by the imagery in winter and locations of the dominated species in China.
Photograph was taken on 2009/08/09.
The acquisition date of this imagery was 2013/01/26</td></tr>
<tr><td>ENLE</td><td>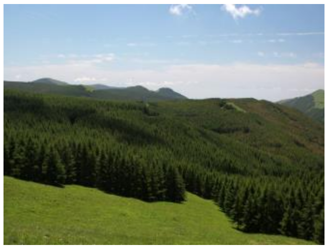</td><td>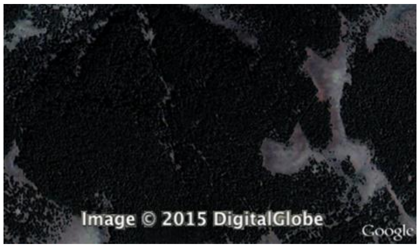
</td><td>Located in Ulanqab, Inner Mongolia
(41.115°N, 112.085°E)
It has dark green and black objects on the surface which are evergreen trees in late May. We identify if the forest is coniferous by the physical photograph and locations of the dominated species in China.
Photograph was taken on 2013/07/05.
The acquisition date of this imagery was 2013/05/14.</td></tr>
<tr><td>GRAS</td><td>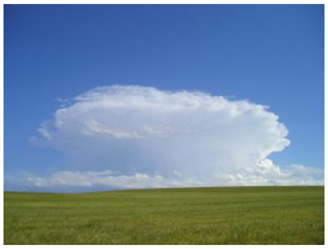</td><td>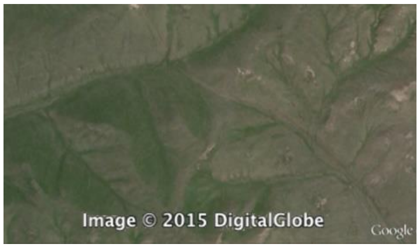
</td><td>Located in Hulunber, Inner Mongolia
(49.585°N, 119.736°E)
It has smooth surface and emerald green color in growing season.
Photograph was taken on 2005/08/19.
The acquisition date of the imagery was 2014/05/30.</td></tr>
<tr><td>OPSH</td><td>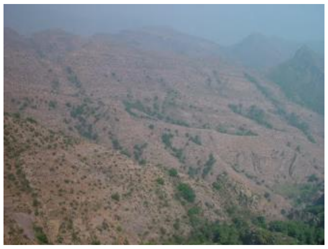</td><td>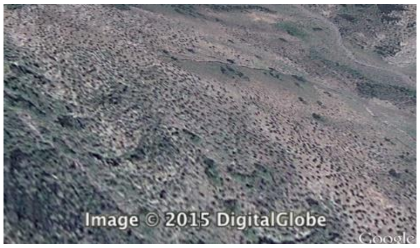
</td><td>Located in Hohhot, Inner Mongolia
(40.810°N, 110.953°E)
It has brown color, texture of mountain with a lot of dots, which are shrubs. The big dots have big canopy. The small ones have small canopy.
Photograph was taken on2006/08/26.
The acquisition date of the imagery was 2011/08/12.</td></tr>
<tr><td>URBN</td><td>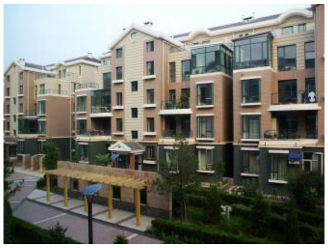</td><td>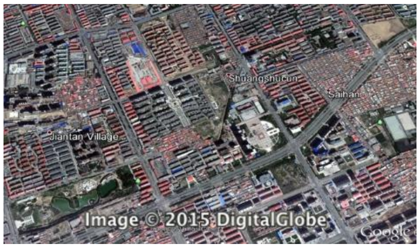
</td><td>Located in Hohhot, Inner Mongolia
(40.791°N, 111.690°E)
It includes a lot of buildings, urban vegetation and roads. It is easy to identity because of human construction.
Photograph was taken on 2008/07/09.
The acquisition date of GE imagery was 2009/08/23.</td></tr>
<tr><td>WATR</td><td>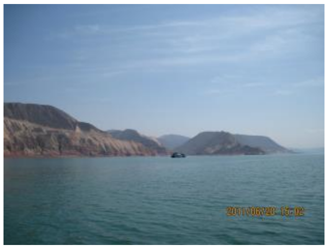</td><td>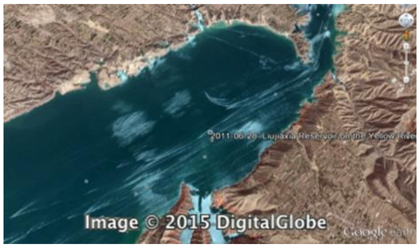
</td><td>Located in Linxia, Gansu
(35.859°N, 103.270°E)
It has a very smooth surface, specific shape and dark blue color (depends on images, sometimes it is green or grey). It can be identified by the shape, color of the water body.
Photograph was taken on 2011/06/28.
The acquisition date of GE imagery was 2010/05/01.</td></tr>
</table>
